# Genome-Wide Identification and Expression Analysis of *WNK* Kinase Gene Family in *Acorus*

**DOI:** 10.3390/ijms242417594

**Published:** 2023-12-18

**Authors:** Hongyu Ji, You Wu, Xuewei Zhao, Jiang-Lin Miao, Shuwen Deng, Shixing Li, Rui Gao, Zhong-Jian Liu, Junwen Zhai

**Affiliations:** 1 Key Laboratory of National Forestry and Grassland Administration for Orchid Conservation and Utilization, College of Landscape Architecture and Art, Fujian Agriculture and Forestry University, Fuzhou 350002, China; ji_hy13@163.com (H.J.); 15980280917@163.com (Y.W.); zxw6681@163.com (X.Z.); dramamiao@163.com (J.-L.M.); 18065177119@163.com (S.D.); 18806006077@163.com (S.L.); 13961833825@163.com (R.G.); 2 College of Forestry, Fujian Agriculture and Forestry University, Fuzhou 350002, China

**Keywords:** *Acorus*, *WNK* gene family, expression pattern, abiotic stress

## Abstract

WNK (With No Lysine) kinases are members of serine/threonine protein kinase family, which lack conserved a catalytic lysine (K) residue in protein kinase subdomain II and this residue is replaced by either asparagine, serine, or glycine residues. They are involved in various physiological regulations of flowering time, circadian rhythms, and abiotic stresses in plants. In this study, we identified the *WNK* gene family in two species of *Acorus*, and analyzed their phylogenetic relationship, physiochemical properties, subcellular localization, collinearity, and *cis*-elements. The results showed twenty-two *WNK*s in two *Acorus* (seven in *Ac. gramineus* and fifteen in *Ac. calamus*) have been identified and clustered into five main clades phylogenetically. Gene structure analysis showed all *WNK*s possessed essential STKc_WNK or PKc_like superfamily domains, and the gene structures and conserved motifs of the same clade were similar. All the *WNK*s harbored a large number of light response elements, plant hormone signaling elements, and stress resistance elements. Through a collinearity analysis, two and fourteen segmental duplicated gene pairs were identified in the *Ac. gramineus* and *Ac. calamus*, respectively. Moreover, we observed tissue-specificity of *WNK*s in *Acorus* using transcriptomic data, and their expressions in response to salt stress and cold stress were analyzed by qRT-PCR. The results showed *WNK*s are involved in the regulation of abiotic stresses. There were significant differences in the expression levels of most of the *WNK*s in the leaves and roots of *Acorus* under salt stress and cold stress, among which two members in *Ac. gramineus* (*AgWNK3* and *AgWNK4*) and two members in *Ac. calamus* (*AcWNK8* and *AcWNK12*) were most sensitive to stress. In summary, this paper will significantly contribute to the understanding of *WNK*s in monocots and thus provide a set up for functional genomics studies of *WNK* protein kinases.

## 1. Introduction

Protein kinases are a class of intracellular messenger-dependent enzymes that mediate and amplify protein phosphorylation and aid in signaling [1], and these enzymes catalyze the translocation of γ-phosphate from ATP or GTR to specific serine/threonine, tyrosine (Tyr), or histidine residues of protein substrates [2,3,4]. WNK (With No Lysine) kinase was a newly discovered silk/threonine protein kinase in recent years, which was successfully cloned and isolated for the first time in the plant *Arabidopsis thaliana* in 2002 [5]. The WNK gene family encodes a special type of serine/threonine protein kinase, with a lysine replaced by a cysteine at the key site of the active center of its substructural domain, which is highly conserved among the other protein kinases, and this unique feature distinguishes the *WNK* kinases as a distinct class of protein kinases [6,7].

In plants, WNK kinases are involved in a variety of physiological processes, particularly in the regulation of circadian rhythms to control photoperiodic responses and maintain key physiological cycles [8,9]. However, studies on the function of *WNK*s in plants are scarce, and existing studies are focused on *Arabidopsis*, with more detailed studies on *AtWNK1* and *AtWNK8* [5,10,11]. The *AtWNK1*, *AtWNK2*, *AtWNK4*, and *AtWNK6* may play an important role in the regulation of circadian rhythms in *Arabidopsis* [5], in which *AtWNK1* was shown to interact with *APRR3* and phosphorylate the APRR3 component of the clock-related APRR1/TOC1 quintet in plant biological clock-controlled circadian rhythms [5,12]. And, the transcript levels of several genes in the photoperiod pathway for flowering, such as *ELF4*, *TOC1*, *CO* and *FT*, were altered in *AtWNK* mutants, suggesting the *Arabidopsis WNK* gene family regulated flowering time by modulating the photoperiod pathway [11]. In addition, Hong–Hermesdorf et al. [13] found the *AtWNK8* protein kinase was able to bind and phosphorylate vacuolar H+-ATPase subunit C (VHA-C) at multiple sites through yeast two-hybrid experiments, and *AtWNK8* participated in the intricate regulation of ion transport processes in plants by interacting with the C-terminus of this subunit. *AtWNK9* plays a positive role in ABA signaling cascade and enhances drought tolerance in transgenic *Arabidopsis* [14]. The soybean *GmWNK* regulates gene tissue-specific and stress-responsive expression in root architecture [15,16], the *OsWNK* has a major role in the root formation and architecture [17]. The *OsWNK1* expression pattern was observed and suggested its involvement in the regulation of biological circadian cycle and its possible role in abiotic stresses [18].

Acorales is sister to all other monocots and contains only one family, Acoraceae, and just one genus with two species *Acorus gramineus* Solander ex Aiton and *Acorus calamus* Linnaeus [19], which have important ecological and ornamental value. Because of its unique phylogenetic position, comparative analysis with other angiosperms could yield important insights into its growth and development. Recently, *Acorus* genomics have revealed the molecular mechanisms underlying the formation of key traits such as vascular cambia, secondary xylem development, and cotyledon evolution in *Acorus* [20], but no reports have been made on the mechanisms of *WNK*s regulation in *Acorus*.

In this study, we chose the diploid species *Ac. gramineus* and the tetraploid species *Ac. calamus* for the identification of *WNK*s and analyzed the basic characteristics of *WNK*s in *Acorus* by using a bioinformatics analysis method, such as gene structure, motif composition, chromosomal localization, and phylogenetic tree analysis. We also analyzed their specific expression in different tissues based on transcriptome data combined with qRT-PCR to analyze their expression changes under salt and cold stresses. The results provide useful information on the biological functions of *WNK*s in *Acorus* and emphasize the functional importance of *AgWNK*s and *AcWNK*s in different tissues.

## 2. Results

### 2.1. Identification and Physicochemical Properties of the Acorus WNKs

Using eleven *Arabidopsis WNK* protein sequences as the queries, twenty-two *WNK*s of *Acorus* (seven in *Ac. gramineus* and fifteen in *Ac. calamus*) were identified by BLAST searches and HMMER. The 22 *WNK*s of *Acorus* were named *AgWNK1–7* and *AcWNK1–15* according to the distribution order of the genes on the chromosome, respectively. 

The physicochemical properties of *Acorus* were analyzed by the ExPasy online tool. These *WNK* protein sequences varied considerably in the number of amino acids (AA), ranging from 227 to 772 aa, with an average length of 554 aa (Table 1). The protein molecular weights (MW) ranged from 25.58 to 86.28 kDa, and the average molecular weight was 62.63 kDa. The isoelectric point (pI) ranged from 4.58 to 8.16, with two *WNK*s having an isoelectric point greater than 7.0, making them alkaline, and twenty *WNK*s having an isoelectric point less than 7.0, making them acidic. The instability indexes (II) range from 33.00 to 52.28, and the aliphatic indexes (AI) range from 68.61 to 92.90. The deduced grand average of hydrophilic (GRAVY) values were less than 0, which indicated all *WNK*s of *Acorus* were hydrophilic (Table 1). In addition, the predicted subcellular localization showed most *WNK*s were distributed in the nucleus, and a few *WNK*s were distributed in the cytoplasm, suggesting the *WNK*s may have different functional roles.

### 2.2. Phylogenetic Analysis

**Figure 1:**
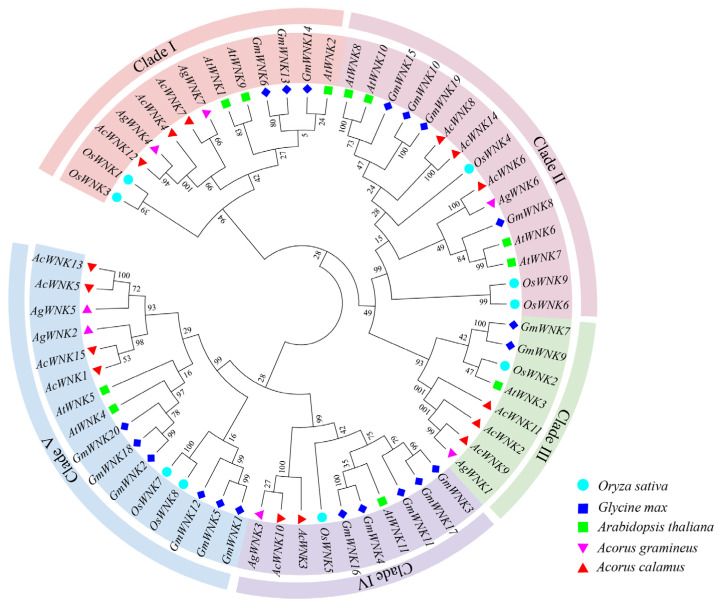
Phylogenetic tree of *WNK*s based on the *WNK* protein sequences of *Ac. gramineus*, *Ac. calamus*, *A. thaliana*, *O. sativa,* and *G. max*.

To reveal the evolutionary relationship of the *Acorus WNK* gene family and help with its classification, an evolutionary tree was constructed with sixty-two *WNK*s from *Ac. gramineus* (seven *WNK*s), *Ac. calamus* (fifteen *WNK*s), *A. thaliana* (eleven *WNK*s), *Oryza sativa* (nine *WNK*s), and *Glycine max* (twenty *WNK*s) (the protein sequences in Supplementary Table S1). Based on the topology of the phylogenetic tree, the *WNK* gene family can be divided into five clades, namely clade I, II, III, IV, and V. In all evolutionary branches, the *WNK*s of monocots and dicots can be divided into two sub-branches with high bootstrap values, such as in Clade I, the dicot plants *GmWNK*13, 6, 14 and *AtWNK*1, 9, 2, and the monocot plants *AcWNK4*, 7, 12 and *AgWNK*4, 7 are divided into two sub-branches (Figure 1). These results revealed the classification and evolutionary relationships of the *WNK* gene family in *Acorus*.

### 2.3. Protein Conservative Domain and Gene Structure Analysis

**Figure 2:**
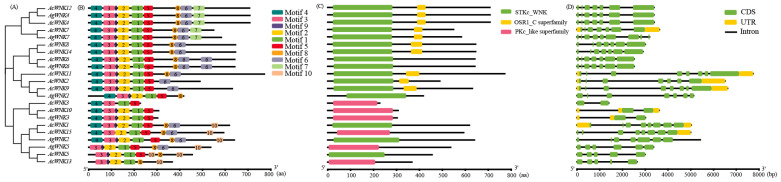
Phylogenetic relationships, motif, and structure of *WNK*s in *Acorus*. (**A**) Phylogenetic relationships among *WNK*s. (**B**) Distribution of conserved protein motifs of *WNK*s. (**C**) Predicted conserved protein domains of *WNK*s. (**D**) Exon-intron structures of *WNK*s.

To further understand the gene structure of *Acorus*, the conserved protein motifs were analyzed on the MEME website and set as Motif 1–Motif 10. The results showed most of the conserved motifs of the *WNK*s existed in the N-terminal domain and were arranged in the order of Motif 4, Motif 3, Motif 9, Motif 2, Motif 1, Motif 5, Motif 8, Motif 6, Motif 7, and Motif 10 (Figure 2B). All *WNK*s possessed essential STKc_WNK or PKc_like superfamily domains, including motifs 4, 3, 9, 2, 1, and 5 (Figure 2C). In the branches of Clades I, II, and III, except for *AgWNK6*, *AcWNK6,* and *AcWNK1*, the remaining *WNK*s also contained an additional OSR1_C superfamily domain (Figure 2C). By analyzing the intron-exon structure, it was found noticeable variations in the number of exons were observed among the *WNK*s, with most members containing five to nine exons, while the three members in Clade IV had only two exons (Figure 2D). The analysis of conserved motifs further supported the phylogenetic relationships and classification of *WNK*s in *Acorus* (Figure 2A).

### 2.4. Collinearity and Location Analysis on Chromosomes

The results showed the *WNK*s of *Acorus* were distributed on different chromosomes. The seven *WNK*s were distributed on six chromosomes in *Ac. gramineus*, Chr 05–09 and Chr 12. The Chr 05 contained two *WNK*s, while the other chromosomes each contained one *WNK*. The fifteen *WNK*s were distributed on eleven chromosomes in *Ac. Calamus*, Chr 03–08, Chr10, and Chr 16–21. The Chr 10 and Chr 20 contained two *WNK*s, while the other chromosomes each contained one *WNK* (Figure S1).

**Figure 3:**
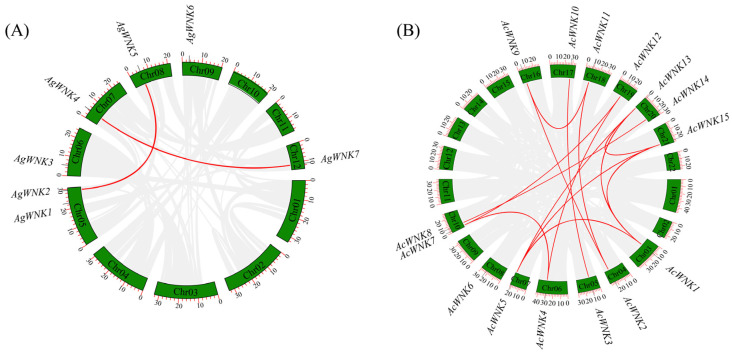
Synteny analysis of the *WNK*s in the two *Acorus* species. (**A**) Synteny analysis of *AgWNK*s. (**B**) Synteny analysis of *AcWNK*s. Red lines represent segmental duplicated gene pairs.

In addition, the collinear relationship of the *WNK*s of *Ac. gramineus* and *Ac. calamus* was analyzed to identify potential replication events in the evolution of the *WNK*s in *Acorus*, respectively. The results showed most of the *WNK*s in *Acorus* had a collinear relationship (Figure 3). And, two and fourteen segmental duplication events were identified in the *Ac. gramineus* genomes and *Ac. calamus* genomes, respectively.

### 2.5. Cis-Elements Analysis

**Figure 4:**
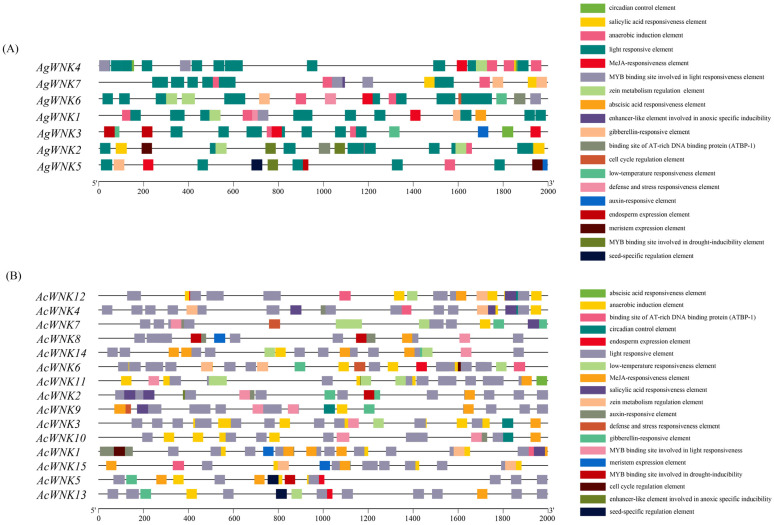
*Cis*-element analysis of WNK promoters in the *Acorus* species. (**A**) The *cis*-element of *Ac. gramineus*. (**B**) The *cis*-element of *Ac. calamus*.

In this study, the 2000 bp upstream sequences of *Acorus WNK*s and *cis*-elements were analyzed by PlantCARE. A total of 19 types *cis*-elements were identified in the promoter regions of *WNK*s in *Acorus* (Figure 4). Among the identified elements, the number of light-reaction related elements is the highest, with 92 (about 53% of the total) in *Ac. gramineus* and 184 (about 47% of the total) in *Ac. calamus*. The *cis*-regulatory elements associated with hormones were widely distributed, including auxin response, salicylic acid response, gibberellin response, abscisic acid response, and MeJA response, with 39 (about 22% of the total) in *Ac. gramineus* and 109 (about 28% of the total) in *Ac. calamus* (Figure 4 and Figure S2, and Supplementary Table S2). Significantly, stress-related *cis*-elements were equally widely distributed, including defense and stress response, drought induction, anaerobic induction, and low-temperature response, with 23 (about 13% of the total) in *Ac. gramineus* and 63 (about 16% of the total) in *Ac. calamus* (Figure 4 and Figure S2, Supplementary Table S2). In addition, other identified elements in the promoters included endosperm expression elements, zein metabolism regulation elements, and meristem expression elements. Changes in type and quantity of elements suggested the wide functional variability of *WNK*s in *Acorus*.

### 2.6. Expression Pattern of WNKs

**Figure 5:**
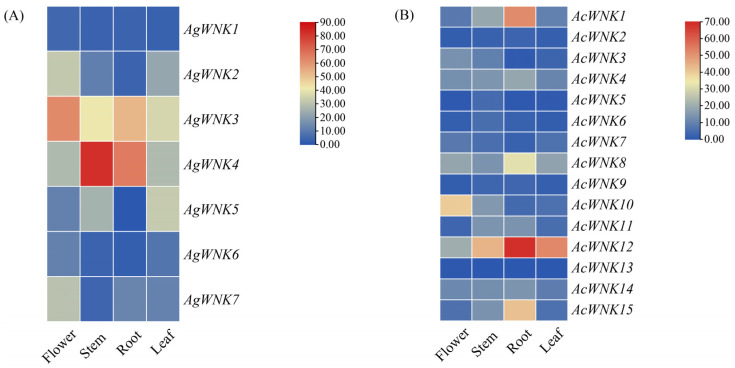
Expression analysis of *WNK*s in different tissues. (**A**) Expression pattern of *WNKs* in *Ac. gramineus*. (**B**) Expression pattern of *WNKs* in *Ac. calamus*.

The majority of *WNK*s showed low expression levels across tissues, while *AgWNK4* and *AcWNK12* exhibited relatively high constitutive expression levels in all tissues. And, the expression of *AcWNK12* in roots and *AgWNK4* in stems was the highest. Importantly, several *WNK*s displayed high expression levels in specific tissues, for instance, *AgWNK4*, *AgWNK3*, *AcWNK12*, *AcWNK1,* and *AcWNK15* in roots, *AgWNK4* and *AcWNK12* in stems, and *AgWNK12* in leaves (Figure 5 and Supplementary Table S3). These findings suggested different *WNK*s may play specific roles in particular tissues.

### 2.7. qRT-PCR Analysis

**Figure 6:**
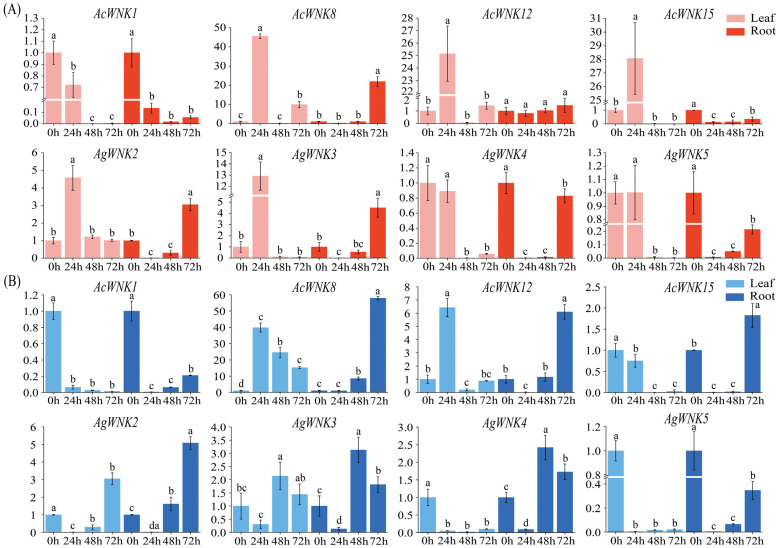
Real-time reverse transcription quantitative PCR (RT-qPCR) validation of eight *WNK*s under salt stress (**A**) and cold stress (**B**). X-axis represents processing time; Y-axis represents relative expression values (2^−∆∆CT^). Following analysis of variance, significant differences identified by Duncan’s test (*p* < 0.05), using SPSS v.25 are represented by different letters.

To explore the expression pattern of the *WNK*s under salt stress and cold stress, we performed real-time quantitative PCR (RT–qPCR) on eight *WNK*s of *Acorus*. The results showed the expression of most *WNK*s in leaves was up-regulated after salt stress, while the expression of these eight *WNK*s in roots was significantly down-regulated (Figure 6). Under 200 mM NaCl treatment, *AcWNK8*, *AcWNK12*, *AcWNK15*, *AgWNK2*, *AgWNK3*, and *AgWNK5* were significantly up-regulated more than five-fold in leaves, and *AcWNK8* expression was up-regulated as high as forty-five-fold and then down-regulated, while the expression of *AcWNK1* and *AgWNK4* was significantly down-regulated in leaves. In roots, the expression of each *WNK* showed a trend of down-regulation followed by up-regulation. Unlike the results of salt stress, the expression of most *WNK*s showed different degrees of down-regulation in both leaves and roots under cold stress. Under 4 °C treatment, only the expression of *AcWNK8* and *AcWNK12* was significantly up-regulated in leaves, while the expression of other *WNK*s was significantly down-regulated by 0.5–1-fold. In roots, the expression of each *WNK* showed a trend of decreasing and then increasing, and it was noteworthy the expression of *AgWNK3* and *AgWNK4* decreased significantly at 24 h, increased significantly at 48 h, and decreased again at 72 h. In conclusion, these *WNK*s were expressed in both roots and leaves under 200 mM salt stress and 4 °C cold stress, suggesting the *WNK*s of *Acorus* were responsive to both salt stress and cold stress.

## 3. Discussion

With no lysine (*WNK*) are soluble serine/threonine protein kinases, and they are so called due to the unusual location of an important catalytic lysine. They can be activated by upstream signals through phosphorylation and regulate the activity of downstream target substrates, serving as important regulators in cellular physiological processes. The distribution of *WNK* is restricted to higher multicellular organisms, and related research is focused on humans and animal [22]. The *WNK* was first cloned and characterized in Rattus norvegicus [6]. Compared to animals, plants have a greater number of *WNK*s [11]. Eleven *WNK*s were successfully identified and isolated in *Arabidopsis* [5], nine *WNK*s were found in rice [17], and twenty-six *WNK*s were identified in soybean [16], which was significantly higher than the previously available results [15]. In contrast, the information about *WNK* family members in *Acorus* is not clear, and their evolutionary relationships are unknown. Therefore, it is necessary to carry out further studies in this area.

Genome duplication provides the original genetic material for biological evolution and is extremely important for plant evolution [23]. A total of twenty-two *WNK*s were identified in *Acorus*, of which seven in *Ac. gramineus* and fifteen in *Ac. calamus*, significantly more than *Arabidopsis* [11] and rice [17]. Interestingly, we found the number of *WNK*s in the allotetraploid *Ac. calamus* was two-fold higher than that of the diploid *Ac. gramineus*, as in the case of soybeans, suggesting *Acorus* underwent a whole genome duplication (WGD) event, resulting in a highly duplicated genome and an increased number of *WNK*s [16,20,24]. By constructing a phylogenetic tree of *Ac. gramineus*, *Ac. calamus*, *A. thaliana*, soybean, and rice, the *WNK*s were divided into five clades. And, based on the phylogenetic clustering of these kinases, *WNK*s clustered in the same clade may have similar functions. Meanwhile, we found paired *WNK*s had similar gene structures and high internal node bootstrap values (100%), such as *AcWNK8/14* and *AcWNK5/13*, suggesting there may be parallelism and functional redundancy in these genes, which may be caused by gene duplication events.

The structural domain analysis, gene structure, and subcellular localization of the 22 *WNK* gene family members in *Acorus* suggested the number of amino acids (AA) of *Acorus WNK* gene family, ranging from 227 to 772 aa, was similar to that of *Arabidopsis*, rice, and soybean [11,15,16,17]. Prediction of gene structure of *Acorus* indicated the *WNK*s consisted of two to nine exons, consistent with the results for soybean [16]. Interestingly, we observed almost each sub-clade had equal numbers of exons and introns in their genomic structure. The predicted subcellular localization showed *WNK*s were mainly localized in nucleus, and a few in chloroplast, cytoplasm, and cytoskeleton. This result was consistent with the prediction for rice in mPLoc, whereas the prediction for rice in mPLK showed all *OsWNK* proteins were located in the cytoplasm [17], and the prediction for soybean *GmWNK* proteins were locations in different cellular compartments [16].

Members of the *WNK* kinase family share a common feature, namely the absence of a very conserved catalytic lysine residue in the functional structure of the kinase, essential for the maintenance of protein kinase activity. In plants, this conserved lysine residue is replaced by a serine. Subdomain I, at the NH2 terminus of the kinase domain, contains the consensus motif Gly-X-GIy-X-X-Gly-X-Val, but in *WNK* kinase the third glycine is replaced by a lysine (K) residue, which alters the consensus motif to Gly-X-Gly-X-X-Lys-X-Val. As in rice and soybean, the conserved structural domains of the *WNK* gene family members of *Ac. gramineus* and *Ac. calamus* showed similar changes [16,17]. Predicting conserved domains in *AgWNK* and *AcWNK* gene members revealed the presence of N-terminal protein kinase domains classified as STKc_WNK and PKc_like superfamily, with a distinctive positioning of the catalytic lysine [10,25]. In addition, in Clade I–III, all *WNK*s (except *AcWNK6*, *AgWNK6,* and *AgWNK1*) possess an additional oxidative-stress-responsive kinase 1 C-terminal domain (OSR1 domain), approximately 40 amino acids in length, involved in the signaling cascade that activates the Na/K/2Cl cotransporter during osmotic stress [26].

About the *WNK* genes family function studies, the focus has mainly been on humans and animals. In mammals, *WNK*s are mainly involved in intercellular ion transport [27], whereas fewer studies are conducted on the function of *WNK*s in plants, and relevant studies focus on a few model plants. *WNK* has been shown to be mainly involved in the regulation of circadian rhythm and interaction with V ATPase C subunit in *A. thaliana* [12,13]. *WNK* with might be involved in regulation of various abiotic stresses in rice and soybean [16,17]. *Cis*-elements play a crucial role as regulatory factors in hormone responses and resistance to various stresses during plant growth and development [28]. Predicting the promoter regions of all *WNK*s revealed the presence of diverse *cis*-elements, involved in light response, plant hormone signaling, and stress resistance. The research results showed the number of light-responsive *cis*-elements was the largest in *WNK* promoters, suggesting their potential involvement in *Acorus* photoperiodic response or circadian rhythm [5], consistent with the results of *Arabidopsis* and soybean [11,16], where the *WNK* gene family regulates plant flowering time through the photoperiod pathway. The second most numerous *cis*-elements were jasmonic acid and abscisic acid, indicating a close relationship between the *WNK* gene family and stress response in plants. Related studies have indicated jasmonic acid and abscisic acid play important roles in adversity stress responses (such as plant drought, high salt, high temperature, etc.) in addition to regulating related physiological processes in plants and inducing plant resistance to these stressors [29,30,31]. The expression pattern of a gene can directly affect its regulatory function. In our study the majority of *WNK*s showed low expression levels across tissues, while several *WNK*s displayed high expression levels in specific tissues. For instance, the expression of *AcWNK12* in roots and *AgWNK4* in stems was the highest. At the same time, the frequent occurrence of abscisic acid-responsive element and the jasmonic acid element in the promoters indicated the up-regulation of *WNK* expression in roots and stems may be closely related to plant stress responses. They serve as initial clues for further investigation and functional characterization of *WNK*s in different physiological processes in plants [32,33].

*WNK* kinases possess kinase activity and undergo autophosphorylation, which plays a crucial role in stress-induced signal transduction pathways [34,35,36]. *WNK*s are involved in responses to abiotic stress. For instance, the *GmWNKs* of soybean are involved in salt stress response [16]; *AtWNK9* of *A. thaliana* is involved in drought stress [14]. In our study, we found the transcript levels of most *WNK*s in *Acorus* were significantly increased in leaves within 24 h after 200 mM salt stress. Notably, the expression pattern of *AcWNK1* and *AgWNK4* was in contrast to the other *WNK*s, with a significant decrease in expression within 24 h in leaves. Similar results were reported in previous studies, where soybean *GmWNK1* [15] and *GmWNK5* [16] were down-regulated after two hours of NaCl treatment, and the overexpression of *GmWNK* altered the plant’s sensitivity to salt stress and osmotic stress. In contrast, the transcript levels of most *WNK*s in *Acorus* decreased and then increased after cold stress, except for *AcWNK8* and *AcWNK12*. Interestingly, the expression of *WNK*s in roots of *Acorus* decreased and then increased significantly in both salt and cold stress within 24–72 h, with the phenomenon of delayed regulatory response. Combined with the results of qRT-PCR and *cis*-elements, the results indicated *WNK*s may play a positive role in jasmonic acid and abscisic acid signaling cascades and enhanced salt and cold tolerance in *Acorus*. However, the specific molecular mechanisms need to be addressed by further research. These results revealed the dynamics and complexity of the expression of *WNK*s in *Acorus* under salt stress and cold stress, and laid the foundation for research on breeding strategies for stress tolerance in monocot plants. However, further research and validation are needed for specific applications.

## 4. Materials and Methods

### 4.1. Data Sources

The genome sequence and annotation files of *Ac. gramineus* and *Ac. calamus* [20] were downloaded from the National Center for Bioinformation Information (NCBI, https://www.ncbi.nlm.nih.gov/, accessed on 10 September 2023) (accession: PRJNA782402), and the *WNK* protein sequence files of *A. thaliana* from the *Arabidopsis* Information Resource (TAIR, http://www.arabidopsis.org/, accessed on 10 September 2023).

### 4.2. Identification and Physicochemical Properties of the WNK Gene Family

The *WNK* protein sequences from *A. thaliana* were used as references to find the *WNK* protein sequences of *Ac. gramineus* and *Ac. calamus* using a local BLASTP search (built-in TBtools v2.019 [21]), and to manually remove the redundant sequences. In addition, uncertain genes were uploaded to the NCBI website (https://blast.ncbi.nlm.nih.gov/, accessed on 10 September 2023) for a BLASTP search.

The online software ExPASy 3.0 (https://web.expasy.org/protparam/, accessed on 13 September 2023) [37] was used to analyze the number of amino acids (AA), molecular weight (MW), theoretical Pi (pI), instability index (II), aliphatic index (AI), and grand average of hydropathicity (GRAVY) of *WNK* in *Ac. gramineus* and *Ac. calamus*. The online tool Cell-PLoc 2.0 (http://www.csbio.sjtu.edu.cn/bioinf/Cell-PLoc-2/, accessed on 13 September 2023) [38] was used to predict subcellular localization.

### 4.3. Phylogenetic Analysis of WNKs

To expand the dataset, *WNK* sequences from *Arabidopsis*, rice, and soybean were collected from various plant genome databases, including TAIR (http://www.arabidopsis.org/, accessed on 10 September 2023), NCBI (www.ncbi.nlm.nih.gov/, accessed on 12 September 2023), and Phytozome (http://www.phytozome.net/, accessed on 12 September 2023). A total of 62 *WNK* sequences were introduced into MEGA 7.0 [39]. Multiple sequence alignment was conducted using the Clustal W program and the maximum likelihood method implemented in MEGA 7.0, with 1000 bootstrap replications. The website of iTOL (https://itol.embl.de/, accessed on 15 September 2023) [40] was used to enhance the evolutionary tree.

### 4.4. Gene Structure and Conserved Motif Analysis

The conserved domains were obtained from NCBI-CDD (https://www.ncbi.nlm.nih.gov/Structure/cdd/wrpsb.cgi, accessed on 12 September 2023) and were subsequently visualized using TBtools software [21]. The online software MEME v5.5.5 (https://meme-suite.org/meme/doc/meme.html, accessed on 12 September 2023) [41] was used to analyze the conserved motifs of *WNK*, and the prediction number was set to ten. The TBtools v2.019 software was utilized to visualize the MEME results [21]. Based on the gff3 file, the gene structure was analyzed using the TBtools.

### 4.5. Chromosomal Localization and Synteny Analysis

The software TBtools v2.019 was used to extract the location information of the *WNK* from the genome file and gene annotation file of *Ac. gramineus* and *Ac. calamus,* and to construct the physical map of the *WNK*s on the chromosome. The One Step MCScanX plugin in TBtools was used to analyze the internal structure of the *WNK*s in *Ac. gramineus* and *Ac. calamus*.

### 4.6. Cis-Acting Regulatory Elements Analysis

The upstream 2000 base pair sequences of the promoter codon were obtained from the genomes of *Ac. gramineus* and *Ac. calamus* by using TBtools. The online software PlantCARE (http://bioinformatics.psb.ugent.be/webtools/plantcare/html/, accessed on 17 September 2023) [42] was used to analyze the *cis*-elements in the promoter region of the *WNK*s. The results of all *cis*-elements were visualized using TBtools [21].

### 4.7. Expression Pattern

The plant materials used in this study were obtained from Fujian Agriculture and Forestry University, Fuzhou, China, and the total RNA was extracted using a FastPure Plant Total RNA Isolation Kit (for polysaccharide- and polyphenol-rich tissues) (Vazyme Biotech Co., Ltd., Nanjing, China). Transcriptome sequencing and library construction were completed by Bgi Genomics Co., Ltd. (Shenzhen, China). Then, Bowtie2 2.2.9 was used to align clean reads from four tissues (flowers, roots, leaves, and stem) to the genome and to calculate the gene expression level via RSEM v1.2.8 [43] to obtain the fragments per kilobase of transcript per million fragments (FPKM) values. Finally, a heatmap representing the expression levels was generated using TBtools based on the FPKM values.

### 4.8. Treatment of Plant Materials

The plant materials used in this study were from Fujian Agriculture and Forestry University, Fuzhou, China. Nine pots of mature *Ac. gramineus* and *Ac. calamus* with the same growth conditions were selected and placed in an artificial climate culture room for one week (photoperiod: 16 h light/8 h dark, temperature period: 15 °C/25 °C) and divided them into three groups equally (group-A, group-B, and group-C). The plants of group-A were contrast samples; the plants of group-B were subjected to salt stress by irrigated with 200 mM NaCl solution in their roots (16 h light/8 h dark, 15 °C/25 °C), and the samples of leaves and roots were collected at 0 h, 24 h, 48 h, and 72 h after the initiation of the stress treatment; the plants of group-C were subjected to cold stress at a temperature of 4°C (16 h of light/8 h of darkness), and the samples of leaves and roots were collected at 0 h, 24 h, 48 h, and 72 h after the initiation of the stress treatment [16,17].

### 4.9. qRT-PCR Analysis

The FastPure Plant Total RNA Isolation Kit (for polysaccharide- and polyphenol-rich tissues) (Vazyme Biotech Co., Ltd., Nanjing, China) was employed for RNA extraction. The Reverse Transcript Kit PrimerScript^®^ RT reagent Kit with gDNA Eraser (TaKaRa, Dalian, China) was used for reverse transcription to synthesize cDNA, which was subsequently diluted 10-fold and stored at −80 °C. Primers were designed using Primer Premier 5 software, and we selected Actin gene (Unigene12762) as the reference gene (Supplementary Table S4) [44]. TB Green^®^ Premix Ex Taq™ II (Tli RnaseH Plus) was used for a qRT-PCR analysis on an ABI 7500 Real-Time System. The RT-qPCR conditions were 20 s at 95 °C in the holding stage, and then 40 cycles of 10 s at 95 °C and 30 s at 60 °C in the cycling stage. The relative expressions of the *WNK*s were calculated using the 2^−ΔΔCT^ method [45]. Three independent replicates were performed for each treatment.

## 5. Conclusions

In the present study, we identified twenty-two members of *WNK* in *Acorus* (seven in *Ac. gramineus* and fifteen in *Ac. calamus*), and divided them into five clades based on their phylogenetic relationships. Members of the same clade had similar gene structures and conserved domains. All the *WNK*s harbored a large number of light response elements, plant hormone signaling elements, and stress resistance elements. In addition, expression profiling and qRT-PCR analysis indicated *WNK*s were involved in the regulation of abiotic stresses, especially *AcWNK8*, *AcWNK12*, *AgWNK3,* and *AgWNK4*. Our observations may further elucidate the significance of functional analysis of *WNK*s in Acorus and contribute towards unraveling their biological roles using a functional genomic approach.

## Figures and Tables

**Table 1:** Characteristics of the *WNK*s from *Ac. gramineus* and *Ac. calamus*.

Name	Gene ID	AA ^1^ (aa)	Mw ^2^ (kDa)	pI ^3^	II ^4^	AI ^5^	Gravy ^6^	CDS ^7^ (bp)	Chromosome Location ^8^	Subcellular Localization ^9^
*AcWNK1*	*KAK1320341.1*	618	70.12	5.75	44.91	68.61	−0.72	1857	Chr03: 12409169–12414188	Nucleus
*AcWNK2*	*KAK1319451.1*	489	55.11	4.87	38.00	92.90	−0.24	1470	Chr04: 22866587–22873092	Nucleus, Cytoplasmic
*AcWNK3*	*KAK1318008.1*	227	25.58	4.96	37.08	76.83	−0.37	684	Chr05: 10903953–10905345	Nucleus, Cytoplasmic
*AcWNK4*	*KAK1313845.1*	708	80.49	5.29	51.57	77.23	−0.50	2127	Chr06: 29489259–29492655	Nucleus
*AcWNK5*	*KAK1311781.1*	455	51.47	8.16	45.07	75.03	−0.55	1368	Chr07: 21106937–21109926	Chloroplast, Nucleus
*AcWNK6*	*KAK1311073.1*	642	72.28	4.64	37.33	86.09	−0.22	1929	Chr08: 22639927–22642439	Nucleus
*AcWNK7*	*KAK1305712.1*	549	62.71	5.02	42.96	80.58	−0.49	1650	Chr10: 2507487–2511112	Nucleus
*AcWNK8*	*KAK1307206.1*	645	72.89	4.84	46.28	83.12	−0.44	1938	Chr10: 6894758–6897759	Nucleus
*AcWNK9*	*KAK1295166.1*	631	70.37	4.88	38.75	80.16	−0.50	1896	Chr16: 5502464–5509085	Nucleus
*AcWNK10*	*KAK1292824.1*	307	34.77	5.71	33.94	76.81	−0.48	924	Chr17: 27872713–27876323	Nucleus, Cytoplasmic
*AcWNK11*	*KAK1290085.1*	772	86.28	5.01	46.89	81.74	−0.51	2319	Chr18: 8858348–8866086	Nucleus
*AcWNK12*	*KAK1287662.1*	707	80.25	5.21	51.54	77.33	−0.50	2124	Chr19: 13748207–13751593	Nucleus
*AcWNK13*	*KAK1286675.1*	367	41.82	7.7	46.09	73.30	−0.59	1104	Chr20: 1424439–1427077	Nucleus, Cytoplasmic, Chloroplast
*AcWNK14*	*KAK1285712.1*	645	72.83	4.89	47.97	82.51	−0.451	1938	Chr20: 25344879–25347778	Nucleus
*AcWNK15*	*KAK1284135.1*	593	67.31	5.5	45.07	70.19	−0.624	1782	Chr21: 25677797–25682792	Nucleus
*AgWNK1*	*KAK1271499.1*	417	46.92	6.29	33	88.8	−0.326	1254	Chr05: 22931422–22936549	Nucleus
*AgWNK2*	*KAK1271813.1*	640	72.85	6.33	47.16	71.92	−0.66	1923	Chr05: 29379837–29385258	Nucleus
*AgWNK3*	*KAK1269333.1*	303	34.40	5.88	35.27	77.82	−0.476	912	Chr06: 5286985–5289995	Nucleus
*AgWNK4*	*KAK1266485.1*	708	80.46	5.29	49.81	76.68	−0.517	2127	Chr07: 4327250–4330632	Nucleus
*AgWNK5*	*KAK1264485.1*	536	60.80	5.68	52.28	76.04	−0.545	1611	Chr08: 5773020–5776384	Nucleus
*AgWNK6*	*KAK1264351.1*	642	72.00	4.58	35.3	84.72	−0.222	1929	Chr09: 3488510–3491022	Nucleus
AgWNK7	KAK1259211.1	583	66.26	4.9	44.54	81.05	−0.475	1752	Chr12: 11253328–11256519	Nucleus

^1^ AA, exhibits amino acid; ^2^ Mw, molecular weight; ^3^ pI, theoretical isoelectric point; ^4^ II, instability index; ^5^ AI, aliphatic index; ^6^ GRAVY, grand average of hydrophobicity; ^7^ CDS, Snapgene is used to calculate the CDS length of genes; ^8^ The location of the gene on the chromosome comes from the gff file; ^9^ subcellular localization predicted by Cell-PLoc [21].

## Data Availability

Data are contained within the article and supplementary materials.

## Supplementary Materials

The following supporting information can be downloaded at: https://www.mdpi.com/article/10.3390/ijms242417594/s1.
