# Supplementary material for: Genome-Wide Identification and Expression Analysis of WNK Kinase Gene Family in Acorus
Source: Int J Mol Sci. 2023 Dec 18;24(24):17594. doi: 10.3390/ijms242417594 (PMC10743480; doi:10.3390/ijms242417594)
Supplement: Supplementary file 1 [file ijms-24-17594-s001.zip › Supplementary Figures.pdf]

Supplementary Figures

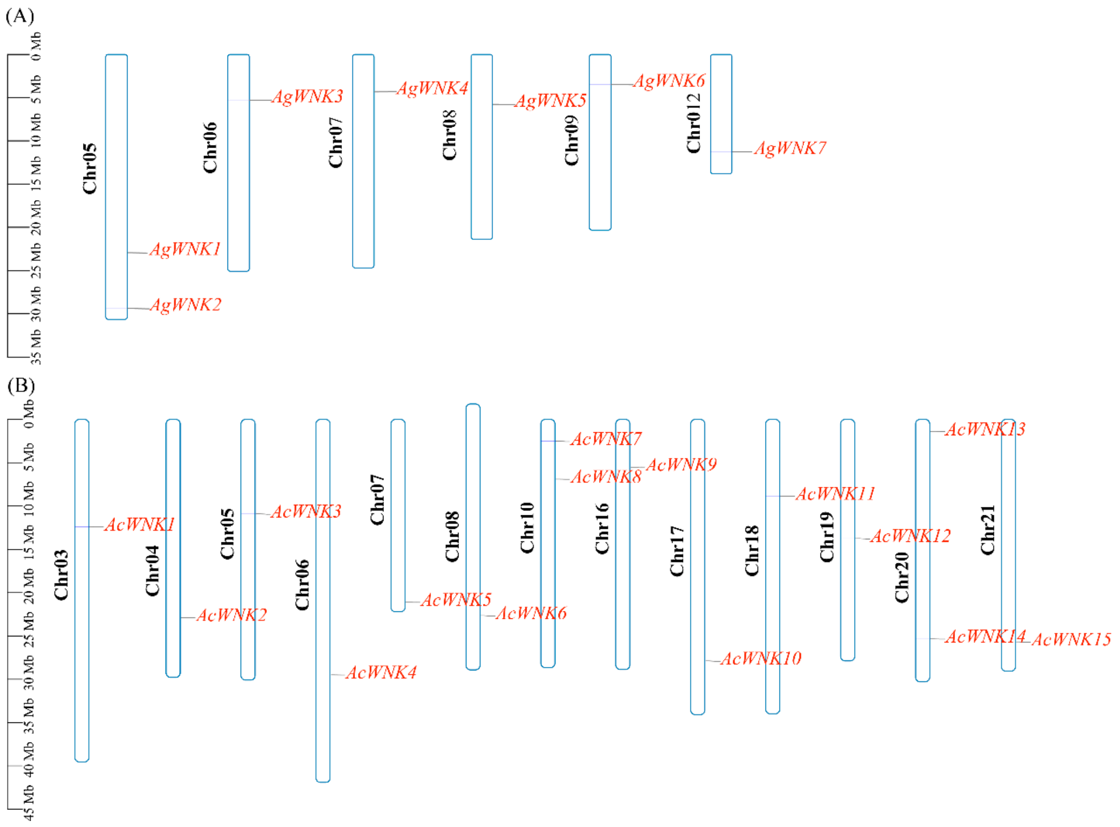

Figure S1. Chromosome distribution in the two *Acorus* species. (**A**) Chromosome distribution in *Ac. gramineus*. (**B**) Chromosome distribution in *Ac. calamus*.

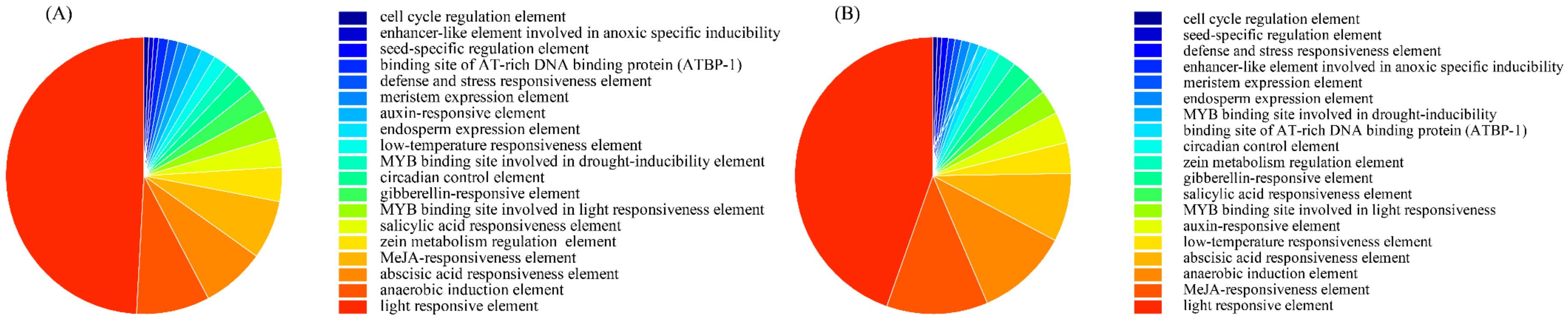

Figure S2 The proportion of various *cis*-elements in *Ac. gramineus* (**A**) and *Ac. calamus* (**B**).
